# Lipid nanoparticles-based RNA therapies for breast cancer treatment

**DOI:** 10.1007/s13346-024-01638-2

**Published:** 2024-06-03

**Authors:** Luigia Serpico, Yuewen Zhu, Renata Faria Maia, Sumedha Sumedha, Mohammad-Ali Shahbazi, Hélder A. Santos

**Affiliations:** 1 Department of Biomaterials and Biomedical Technology, The Personalized Medicine Research Institute (PRECISION), University Medical Center Groningen (UMCG), University of Groningen, Groningen, The Netherlands; 2 Drug Research Program, Division of Pharmaceutical Chemistry and Technology, Faculty of Pharmacy, University of Helsinki, Helsinki, Finland

**Keywords:** Breast cancer, RNA technologies, Lipid nanoparticles, Clinical applications, Gene therapy

## Abstract

Breast cancer (BC) prevails as a major burden on global healthcare, being the most prevalent form of cancer among women. BC is a complex and heterogeneous disease, and current therapies, such as chemotherapy and radiotherapy, frequently fall short in providing effective solutions. These treatments fail to mitigate the risk of cancer recurrence and cause severe side effects that, in turn, compromise therapeutic responses in patients. Over the last decade, several strategies have been proposed to overcome these limitations. Among them, RNA-based technologies have demonstrated their potential across various clinical applications, notably in cancer therapy. However, RNA therapies are still limited by a series of critical issues like off-target effect and poor stability in circulation. Thus, novel approaches have been investigated to improve the targeting and bioavailability of RNA-based formulations to achieve an appropriate therapeutic outcome. Lipid nanoparticles (LNPs) have been largely proven to be an advantageous carrier for nucleic acids and RNA. This perspective explores the most recent advances on RNA-based technology with an emphasis on LNPs’ utilization as effective nanocarriers in BC therapy and most recent progresses in their clinical applications.

**Graphical Abstract:**

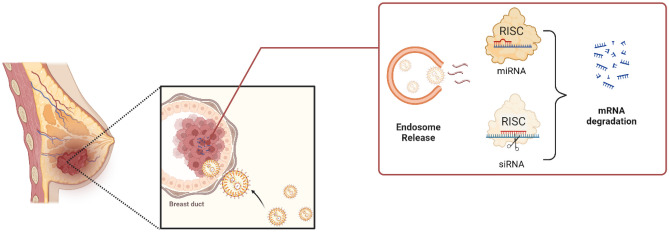

## Introduction

Breast cancer (BC) defines an array of malignancies of the mammary glands. According to GLOBCAN, in 2022, it stood as the most common neoplasm in women, making it one of the most frequently diagnosed cancers worldwide [1]. BC displays high heterogeneity and classifies into four subtypes based on immunohistochemical features: Estrogen receptor positive (ER +); progesterone receptor positive (PR +); human epidermal growth factor receptor positive (HER2 +); and triple negative breast cancer (TNBC) in which none of the previous receptors are expressed [2, 3]. Both ER and PR are diagnostic and prognostic factors. High expression of ER is found in ~ 70–75% of invasive breast carcinomas, and women with an ER + BC show an increased overall survival with respect to the ER- [4]. The ER expression is commonly used to choose the proper treatment because it has predictive value in prognosis and endocrine therapy responsiveness [5]. High levels of PR are a good prognostic factor associated with a better overall survival, while lower levels are generally linked with more severe forms and poor prognosis [6, 7].

HER2 is a protooncogene that encodes epidermal growth factor receptor with tyrosine kinase activity. In BC, HER2 gene is overexpressed in about 20% of invasive forms, and as a prognostic factor, HER2 + is associated with high rates of recurrence and mortality, being a predictive factor for treatment benefits from HER2-targeting agents like trastuzumab, lapatinib, and pertuzumab [8]. TNBC accounts for 10–20% of all breast cancers and  it is characterised by the absence of the markers listed above it is therefore untreatable with target therapies. This, together with the moderate/high aggressiveness and highly proliferative profile makes TNBC associated with the poorest prognosis [9].

**Fig. 1:**
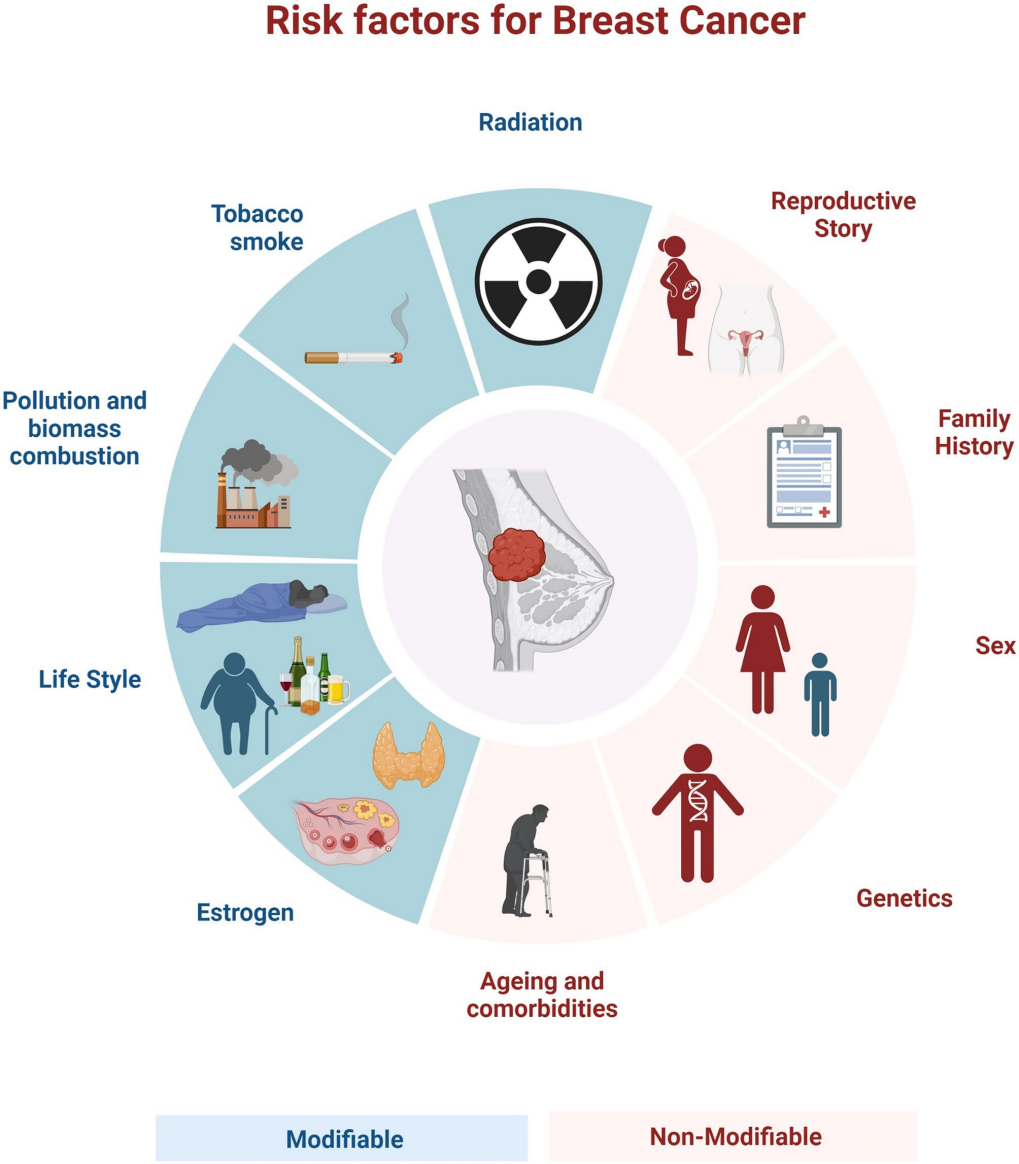
Main risk factors of BC. The blue colour presents the modifiable factors potentially increasing the BC onset and development, while red ones indicate the non-modifiable factors [10–12]. Created with Biorender

BC is marked by several risk factors classified as modifiable and non-modifiable (Fig. 1). Modifiable factors include lifestyle, physical activity, body weight, alcohol consumption, and exposure to artificial light [10, 11]. Non-modifiable factors encompass genetic mutations, sex, age, and family history [12]. Moreover, numerous studies have proved a strong connection between exposure to sex hormones and BC. Events like first menstruation, pregnancy, breastfeeding, menopause and the age of their appearance, as well as their duration, influence the occurrence of BC [13]. The first full-term pregnancy before the age of 30, the number of pregnancies, early menopause are linked to a lower risk of BC [14]. In contrast, early age menarche increases the risk of disease’s development [15].

**Fig. 2:**
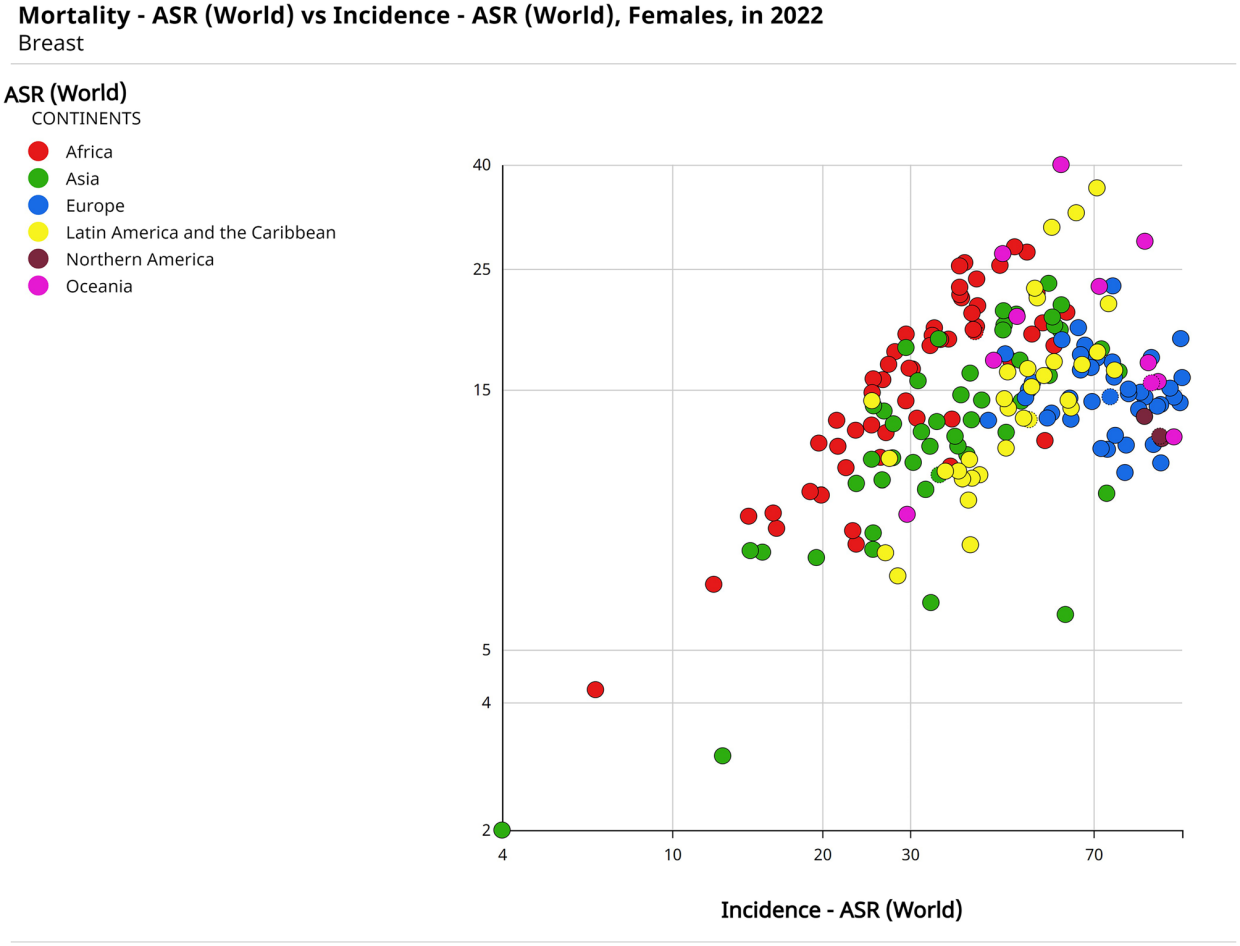
Scatter plot of the incidence and mortality age-standardized rates (ASRs) worldwide, in 2022. The graph shows that the higher rates are reported for Europe and Africa. Source: GLOBOCAN 2022 [16]

Overall BC is a complex disease, where both the combination of genetic susceptibility and the interaction with the environment affect the determination of the phenotype. As reported in the latest WHO epidemiological data report, in 2022, BC represented 23.8% of all cancer cases and 15.4% of cancer-related deaths among women. Globally, this disease caused more than 666,000 fatalities with 2.3 million new cases of BC diagnosed every year [16]. Figure 2 shows the cancer incidence and mortality age standardised rates (ASRs) worldwide, in 2022. The fatality of BC is still high for all the reported countries, with the highest mortality numbers registered in Europe, Africa and Oceania. Moreover, by 2050, the number of cases diagnosed annually is expected to reach 3.36 million, with deaths accounting for 1.06 million yearly. To date, BC remains one of the major burdens on the global healthcare system [17].

The current treatments for BC include surgery, chemo-, radio-, hormone, and immuno- therapy [18]. Patients with resectable lesions undergo surgery, usually followed by pharmacological treatment. When possible, the surgery is breast-conserving, but depending on the spreading of the lesion and its staging, the resection can often imply the complete removal of the breast tissue. Chemotherapy and radiotherapy are still the conventional pharmacological treatments. All the above therapies are often effective and prolong the lifespan of patients [19]. However, they do not prevent risks of recurrence and come with strong side effects, like hair loss, fatigue, increased susceptibility to infections, bone marrow suppression, leucopenia, and anemia [20, 21]. Additionally, drug-specific side effects can also lead to cardiotoxicity and pulmotoxicity [22].

A more recent therapeutic approach is represented by immunotherapy. Cancer immunotherapy harnesses the body's own immune system to target tumor cells, with various approaches [23, 24]; currently, a few of them are also exploited as BC treatment options [25]. These strategies include the use of monoclonal antibodies, immune checkpoint inhibitors, and vaccines designed to prime the immune system against cancer cells [26]. Moreover, over the last decade, adoptive cellular therapies have been implemented, they include the introduction of engineered immune cells into the body to recognise and kill cancer cells [27, 28].

About one third of patients affected by BC exhibit upregulation of HER2 which makes the receptor a good molecular target for selective immunotherapy. Hence, many types of monoclonal antibodies have been developed and demonstrated effectiveness by inhibiting signalling pathways involved in tumor growth [29]. By combining monoclonal antibodies, several pathways may be blocked simultaneously, leading to potential synergism. Among them trastuzumab and pertuzumab − often utilized in combination − are now used in clinical practice for the treatment of those patients [30].

Immune checkpoints are a set of regulatory proteins, crucial in the adaptive immune response. They are essential for maintaining self-tolerance and immune homeostasis. Immune checkpoints also act as immunosuppressors. For example, proteins like programmed cell death protein 1 (PD-1), its ligand (PD-L1), and CTLA-4 (cytotoxic T-lymphocyte-associated protein 4) are exploited by cancer cells to evade the immune system [31]. By blocking these checkpoints with specific inhibitors, it is possible to unleash the immune system's full potential for cancer cell recognition and destruction, leading to improved outcomes for patients [31, 32]. Cancer vaccines are under investigation as a form of active immunotherapy, designed to stimulate the immune system to recognize tumor cells as non-self [33]. As an example, NeuVax™ (a peptide vaccine targeting HER2) is currently undergoing a clinical trial [34]. Furthermore, adoptive cellular therapies involve the manipulation of immune cells obtained from either the tumor or the peripheral blood of patients. These therapies include three main approaches using (I) tumor-infiltrating lymphocytes (TILs), (II) TCR-modified lymphocytes, and (III) chimeric antigen receptor (CAR) T cells [35]. Ongoing clinical trials are investigating the feasibility, safety, and effectiveness of CAR-T cells in targeting HER2, GD2, and CD44v6 surface antigens in BC cells [36]. As an example, the clinical trial Multiple 4SCAR-T Cell Therapy Targeting BC (GIMI-IRB-20005) is currently in phase II, attesting the clinical applicability of this therapeutic platform. Adoptive T-cell therapy holds promise, however, to date, it shows poor functionality and persistence, due to the inter-individual heterogeneity of the T cell population of the tumor microenvironment, inter-tumoral heterogeneity, and resistance mechanisms [37]. Furthermore, high costs and methodologic complexity significantly affect the extensive application of T-cell therapy.

Overall, immunotherapy is not very effective for solid tumors, due to several factors characterising the complex and immunosuppressive cancer microenvironment. Firstly, the dense extracellular matrix (ECM) represents an additional layer, hard to infiltrate. Moreover, the cellular components play a key role in the immune response impairment: myeloid-derived suppressor cells (MDSCs) can prevent specific anti-tumour adaptive immune responses by inhibiting T cells function. In addition, MDSCs produce increased levels of immunosuppressive cytokines like IL-10 and TGF-β [38].

All the above hinders the efficacy of immunotherapy in solid lesions. Moreover, an increased risk of developing autoimmune diseases is a side effect of immunotherapy [39]. Considering all the discussed aspects, novel therapeutic strategies are still needed to obtain a proper treatment of patients affected by BC, especially for the TNBC. The emergence of cancer nano therapeutics opened up many new opportunities by providing some effective tools in cancer treatment, shifting the focus from conventional approaches to personalized medicine [40, 41].

In the last decade, nanoparticles (NPs) have emerged as innovative and promising tools for both diagnostics and therapy; they offer all the physicochemical advantages of nanosystems, and they can be easily modified to suit the desired purpose. As engineered nanomaterials, they proved to be effective drug delivery systems due to their size and their capacity to enter several biological barriers like gastrointestinal walls, blood brain barrier, and cell membranes, overcoming the limitations classically related to traditional drugs [42, 43]. Given the promising characteristics of the NPs, several fabrication strategies and a plethora of materials have been explored to the preparation of new nanosystems for BC treatment [24, 44, 45]. Among them, lipid NPs (LNPs) present major advantages as drug delivery agents by demonstrating precise targeting, reduced side effects, efficient pharmacokinetics and low toxicity, also overcoming the challenge of multidrug resistance [46–50]. Moreover, LNPs production is easily scalable, as demonstrated by several studies and by the massive production of anti-covid vaccines during the last pandemic.

**Fig. 3:**
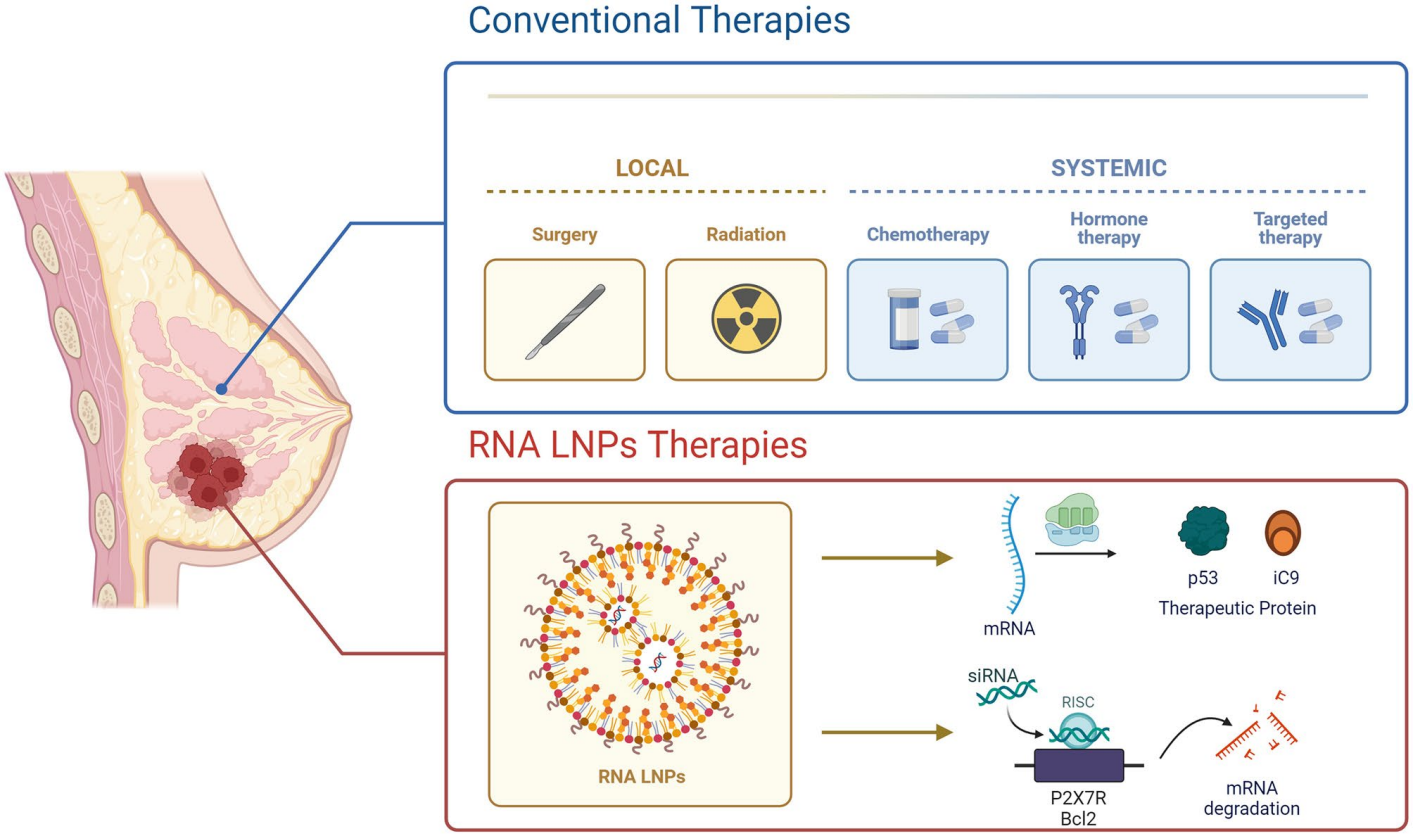
Conventional treatments *vs.* RNA-LNPs therapeutics for BC. Conventional therapies encompass both local interventions, such as surgery and radiation, and systemic treatments, including chemotherapy and hormone therapy. In contrast, RNA-LNP-based therapeutics specifically target tumor sites (Image created with Biorender)

These are just a few reasons why LNPs have been extensively used as delivery platforms, now standing as the most efficient means of RNA delivery [51–55], exemplified by the approval of Patisiran for amyloidosis therapy in 2018 [56, 57]. In the last decade,

RNA-LNPs based therapy has also shown effectiveness in BC treatment (Fig. 3), providing a smart, alternative approach and increasing the success rate of BC cure, including the most resistant forms [56, 58].

But can RNA-LNPs therapy be a standalone approach for BC treatment? This perspective provides an overview of the most recent, mostly within the last 5-years, RNA-based LNPs for BC gene therapy. It focuses on the different types of RNAs used, by giving a perspective on whether RNA-LNPs therapies can be a viable strategy for BC treatment.

## Lipid NPs as drug delivery vehicles

**Fig. 4:**
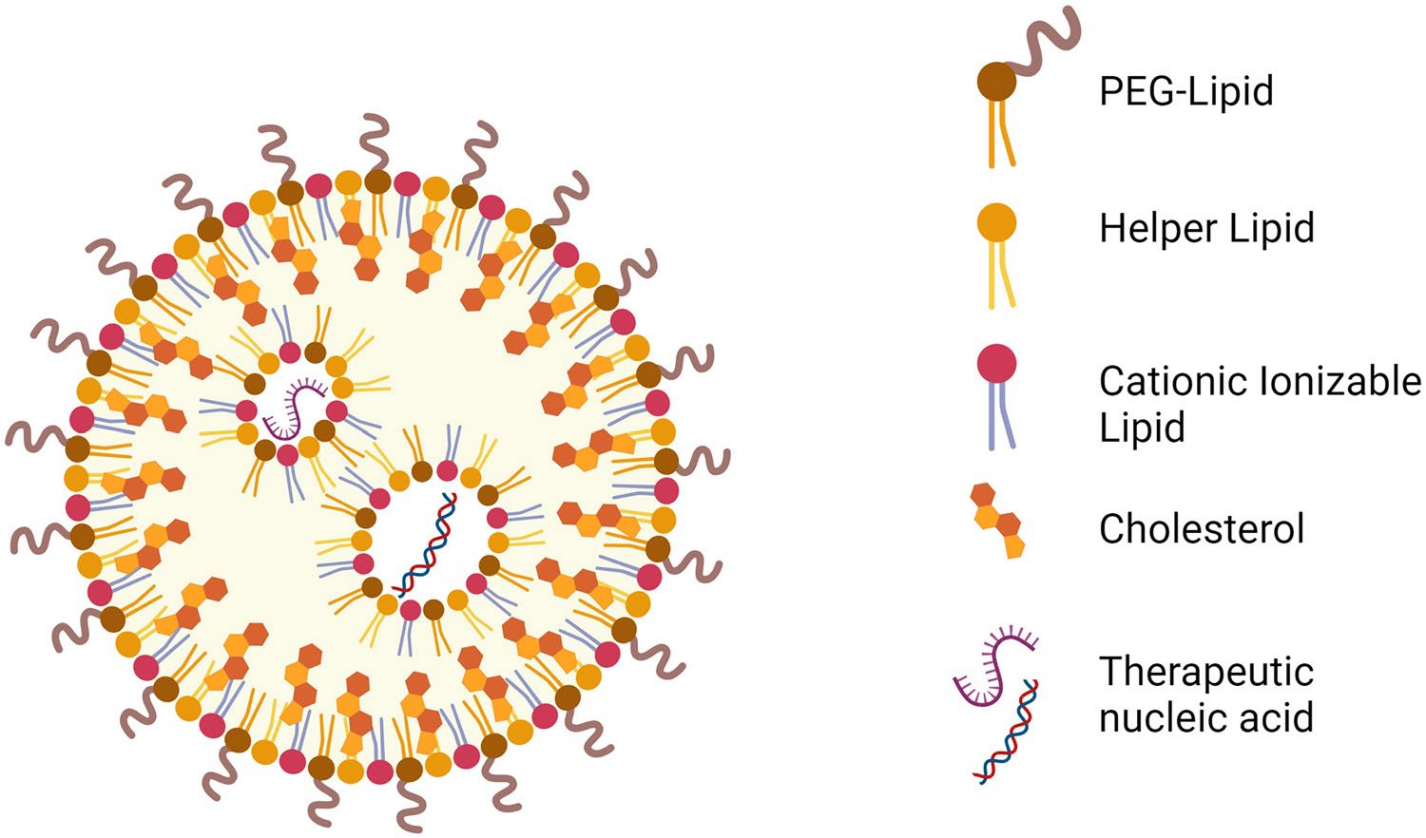
An illustration of RNA-loaded LNP and the lipid components. Image created by Biorender

Over the years, the gradually optimized large-scale production of LNPs successfully moved them from the laboratory to the clinic, making them as pioneer in commercialized nanomedicines. Especially, after the massive administration of Moderna (mRNA-1273) and Pfizer/BioNTech (BNT162b2) COVID-19 vaccines [59–61], LNPs demonstrate their safety and effectiveness as delivery vehicles for mRNAs [62]. The synthesis of LNPs includes two major methods: (1) Bottom-up [63] and (2) top-down approaches [64–67]. The most utilized bottom-up techniques are chemical vapor deposition, laser pyrolysis, microfluidics, and plasma or flame spraying synthesis. Commonly used methods for the top-down fabrication are mechanical milling, electro-explosion, thermal decomposition, chemical etching, nanolithography, etc*.* Therefore, by modulating the synthetic procedures and reaction conditions, desirable alterations in the LNPs' morphological and chemical attributes can be induced [68]. A typical FDA-approved LNP consists of four different types of lipid components. These include ionizable lipids, phospholipids, cholesterol, and PEGylated lipids [69] (Table 1 and Fig. 4). Each lipid component provides the NPs distinctive properties and specific advantages. Therefore, compositions, ratios, and concentrations of lipids, also contribute to obtaining a tailored nanosystem for specific applications.

**Table 1:** List of different functional lipid components and commonly used examples. Adapted from Jung et al*.* (2022) [70]

Lipid Components	Functions	Products
Ionizable lipid	- Effective encapsulation - Biocompatibility - Membrane fusion and endosomal escape - Improvement of transfection efficacy	- ALC-0315 (Pfizer/BioNTech) [71] - SM-102 (Moderna) - DLin-MC3-DMA [72]
Phospholipid	- Structural support - Stable structure (saturated lipids) - Endosome destabilization (unsaturated lipids)	- DSPC (saturated lipid) [73] - DPPC (saturated lipid) [74] - DOPE (unsaturated lipid) [75] - DOTAP [76, 77]
Cholesterol	- Integrity and rigidity - Stable structure - Endosomal release	- Cholesterol - Cholesterol analogues [78, 79] - Cationic cholesterol [80] - Hydroxycholesterol [81]
PEGylated lipid	- Control the size and zeta potential - Steric hindrance - Colloidal stability - Evasion of the mononuclear phagocytic system	- ALC-0159 (Pfizer/BioNTech) [71] - DMG-PEG 2000 (Moderna) [71] - DSPE-mPEG 2000 (Doxil) [82] - DMG-C-mPEG 2000 (Onpattro) [83]

The possibility to modulate the physical–chemical characteristics of LNPs makes them particularly suitable for a stable delivery of small molecules and nucleic acids, contributing to successful cell targeting [69, 84, 85]. LNPs have emerged as efficient tools for advancing the technology for vaccination, immunotherapy, and diagnostics [69, 86]. This is due to the LNPs’ properties like effective stability, high encapsulation efficiency, biocompatibility, limiting off-target effects, protection from the mononuclear phagocyte system (MPS), and other pharmacokinetic and pharmacodynamic attributes [87]. For example, Doxil^®^, the first FDA-approved nano drug [88] has been formulated as a liposomal nanosystem containing doxorubicin. The Doxil^®^ composition contains hydrogenated phosphatidylcholine, cholesterol, and PEG-lipid in a specific ratio (56:39:5) [82, 89]. Since then, research in the development of LNPs has seen an exponential increase. The bibliometric searches conducted in WorldCat indicated that the number of scientific articles on LNPs has exceeded 51,300 in the past three decades (1995–2024). More than 1,900 of these have reported LNPs specifically for the treatment of BC, and to date many undruggable pathways have become treatable thanks to the use of LNPs.

### The effect of structure, composition and surface modification on delivery efficacy

As mentioned above, the structure modulation heavily affects the efficiency of LNPs as delivery system. The inclusion of phospholipids and cholesterol, for example, confers great stability and integrity [65, 66]. The phospholipids, commonly called “helper lipids,” improve the biodistribution of LNPs by binding to specific plasma proteins, thus facilitating cellular uptake and cytosolic entry [90]. In contrast to some cationic lipids, phospholipids are not positively charged and therefore may help reduce inflammation and cytotoxicity potentially induced by cationic lipids [91]. Currently, 1,2-dioleoyl-*sn*-glycero-3-phosphoethanolamine (DOPE) and 1,2-distearoyl-*sn*-glycero-3-phosphoethanolamine (DSPC) [92] are the two most used phospholipids, DSPC has been successfully used in clinical applications, like siRNA therapeutics and the mRNA vaccines against SARS-CoV-2 [93]. DSPC possesses two saturated aliphatic tails, that confer a higher melting point and a solid state at room temperature. This saturation characteristic makes the LNPs membrane composed of DSPC highly rigid and stable. DOPE includes two aliphatic tails, each tail contain a cis-double bond, and when used for gene delivery, it increases transfection efficiency [94]. In addition, the tapered structure of DOPE helps to adjust the size of LNPs and the curvature of the membrane, favouring the cargo encapsulation [95]. Including different helper lipids in the formula of LNPs is expected to achieve the co-delivery of different types of genes. The research of Hamilton et al*.* [28] showed that the encapsulation efficiency of DOPE-incorporated LNPs was significantly improved (88–97%) compared to LNPs containing only DSPC (63%) [96].

Cholesterol plays multiple key roles in LNPs formulations. It can stabilize the surface membrane of LNPs, preventing premature release of nucleic acids by maintaining the integrity of the membrane, and promoting its circulation in the blood to extend the half-life of LNPs [90, 97]. A study by Kim et al*.* showed cholesterol enhanced gene transfection and biodistribution of mRNA-LNPs [98]. Furthermore, cholesterol concentration affects various LNP properties, including particle size, polydispersity, internalization, and overall structure. Very low cholesterol levels can reduce encapsulation efficiency [99]. Conversely, increasing cholesterol levels at the expense of ionisable or cationic lipids result in smaller particle size and reduced polydispersity while maintaining particle integrity [100]. Another study by Kotoucek et al*.* investigated the role of cholesterol in liposome formulations. They found that, when the molar ratio of cholesterol to DSPC was mixed from 0 to 50%, the size of the liposomes also increased. It shows a size-dependent association of liposomes with cholesterol amounts [96]. Therefore, optimizing the ratio between cholesterol and other lipid components is critical to tailor LNP properties to specific formulation requirements.

In the first instance, permanently positively charged lipids such as DOTMA or DOTAP were included in LNPs formulation to facilitate RNA electrostatic interactions with the negatively charged RNA backbone. However, these lipids induce toxicity by destroying cellular and nuclear membranes and by increasing the production of reactive oxygen species [101]. To address this problem, ionizable lipids with pH-sensitive tertiary amines were developed. The ionizable lipids are typically defined by the presence of tertiary amines that are deprotonated at a neutral pH and undergo protonation at lower pHs [63, 69]. Consequently, under lower pH, a positively charged LNPs layer is formed. The latter, in turn, can establish electrostatic interaction with negative charged molecules like nucleic acids [102], allowing an efficient encapsulation. This electrostatic interaction also enhances the biocompatibility of the LNPs, thereby preventing nonspecific binding [103]. The pH in mature endosomes is also acidic [104]. Under such conditions, the protonation of ionisable lipids leads to destabilization of the endosomal membrane, allowing LNPs to escape from the endosome [69]. Notably, the pKa value has proven critical for efficient LNPs delivery and transfection potency. Jayaraman et al*.* firstly identified DLin-MC3-DMA from an amino lipids' library [105], and showed that it has a high transfection efficiency with an optimal pKa value between 6.2 and 6.5. This lipid was later successfully used in Patisiran (Onpattro). In another study, Whitehead identified four key parameters for the efficacy of siRNA delivery in vivo, highlighting the importance of pKa of LNPs containing ionizable lipids [106]. The conclusion of their results showed that the pKa value should be higher than 5.4 to get efficient gene silencing [107].

PEGylated lipids (PEG-lipids) usually represent the smallest portion (typically ∼1.5 mol%) of LNPs. Despite that, these influence several key properties, like size and zeta potential [75]. This directly impacts the delivery efficacy of the LNPs. Usually, PEG with a higher molar mass (20–50 kDa) is used for small-sized drugs. This increases the size of the carrier, which helps them prevent renal clearance. Conversely, larger drugs like antibodies and nanoparticle drugs use a lower molar mass of PEG, like 1 to 5 kDa [108]. These also have roles in preventing the aggregation of LNPs and enhancing the LNPs' blood circulation time. In a very interesting study, Lokugamage et al. [75] demonstrated the PEGylation-mediated stability and long-term distribution of LNPs [75]. They loaded high- and low-PEG LNPs with luciferase to determine their stability. They found that LNPs with a lower mol% of PEG exceeded the quality control cut-off (200 nm in size), indicating the necessity of PEG for delivering high-quality LNPs. Moreover, high PEGylated LNPs showed improved long-term distribution effects, as indicated by their luciferase intensities after 48 h. This proves a lower degradation of luciferase and, consequently, higher stability [109].

In addition, PEGylated lipids are also involved in protecting LNPs through mononuclear phagocyte system (MPS) clearance [102, 110]. Further studies have cumulatively determined their roles in improving encapsulation of nucleic acids, increasing circulatory half-lives, in vivo distribution, and immune response. Conclusively, these decrease fusogenicity and increase the overall stability [85] and bioavailability [103].

Besides structure, surface modification is another key approach in modulating the characteristics of LNPs to specific target cell types. As discussed above, the off-target effect associated with many nanomedicines remains a limitation that needs to be overcome, as it implies an increased dose of drug to achieve the desired therapeutic effect and reduces the bioavailability of the drug. Affinity targeting of the LNPs to the BC lesions can be easily achieved due to several chemical surface modifications [23, 111]. By attaching smart molecules to the available functional groups of the lipids the selectivity and effectiveness can be certainly improved. In a very recent study, Kim et al. engineered LNPs for pTEN mRNA delivery in TNBC cells [112]. They presented a PD-L1-targeting LNP integrating peptides with strong PD-L1 binding affinity onto the LNP surface by conjugating them with PEGylated lipid. This led to an increased affinity for the target, by obtaining an augmented selectivity. These results clearly indicate how the targeting therapy leads to an improved, personalized and more effective treatment.

### Cellular uptake and endosomal escape

**Fig. 5:**
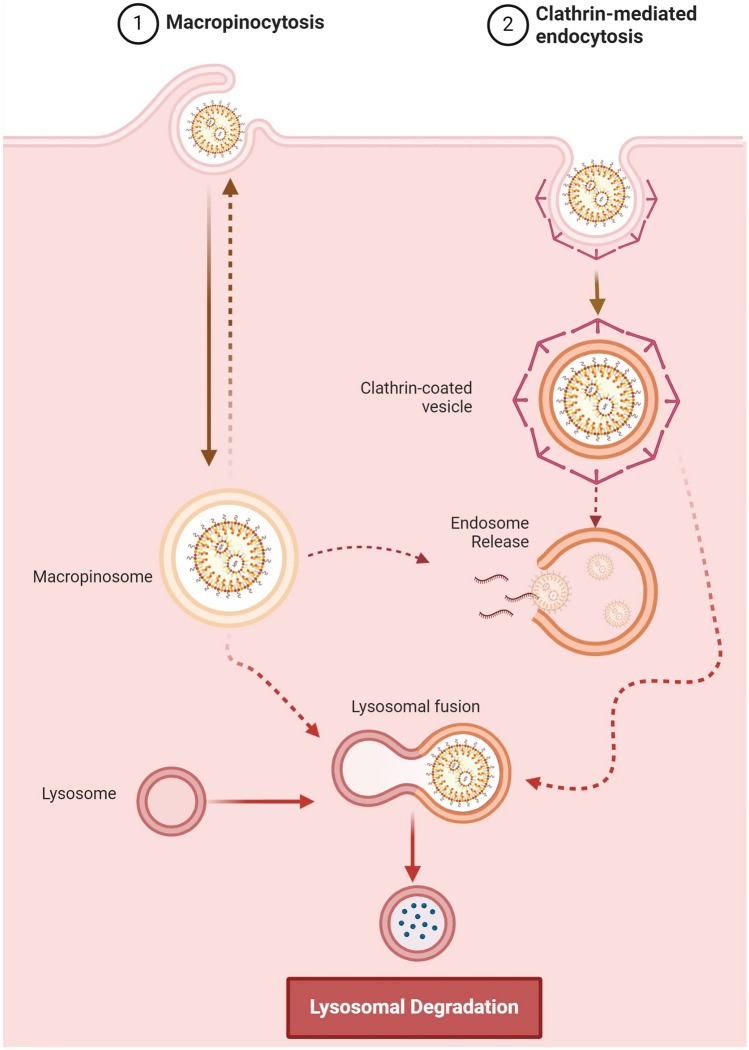
Schematic representation of endocytosis mechanisms for RNA-LNPs. Image adapted from ref [117], and created with Biorender

LNPs overcome several limits of conventional delivery systems, such as low delivery efficiency and immune responses triggered by naked nucleic acids. Currently, LNPs are considered the most effective tool in RNA-based therapies. However, the release into the cytoplasm of loaded nucleic acids is very limited and it still represents an issue. LNPs enter the cell mainly through macropinocytosis and clathrin-mediated endocytosis [84]. Then they are incorporated in early endosomes, which subsequently mature into late endosomes and finally into lysosomes (Fig. 5). Efficient delivery is achieved if the nucleic acid is released from LNPs into the cytoplasm before endosomes become lysosomes, a process known as endosomal escape [113–115]. It is widely acknowledged that modulating the composition of LNPs is a promising strategy to improve endosomal escape and thereby increase transfection efficiency. In a recent work, Tang et al*.* introduced a novel mRNA vaccine based on sialic acid (SA) cholesterol derivatives and cleavable PEG lipid modification, which efficiently targets dendritic cells (DCs) and achieves effective endosomal/lysosomal escape. The SA modification enhances DCs uptake of LNPs by 2 times, facilitates rapid escape from early endosomes (EEs), prevents entry into lysosomes, and promotes mRNA translation in both cytoplasm and endoplasmic reticulum (ER). Additionally, the incorporation of cleavable PEG-lipids enhances cellular uptake and DCs targeting. SA-modified mRNA vaccines exhibit superior efficacy in DCs targeting, enhanced endosomal/lysosomal escape, improved transfection efficiency in DCs, superior tumour treatment effects, and lower side effects compared to commercially formulated mRNA vaccines [116]. In addition, identifying the exact endosomal compartment of escape is also crucial to improve cellular uptake [117] and it still is a debatable aspect. Hence, to understand how the uptake and the transport to endosomal compartments can affect the cargo’s delivery and release, nanocarriers with different properties have been investigated [118–120]. For instance, Paramasivam et al*.* compared the uptake and endosomal distribution of six mRNA-LNPs formulations, with similar size and RNA contents, but diverse chemical composition. The results obtained from different biological assays in primary human adipocytes, fibroblasts, and HeLa cells showed that the LNPs have different uptake efficiencies and endosomal distributions. This demonstrated that multiple mechanisms affect the delivery efficacy, including differences in LNPs composition and surface properties, loaded macromolecules, and their intracellular transport [120].

Overall, the main mechanism of endosomal escape has not yet been clearly determined. However, studying the highly dynamic nature and complex molecular interactions of the endosomal system is critical to clarify the intracellular action site and release kinetics. This would allow to improve the efficacy of LNPs as delivery system of nucleic acid drugs.

## RNA therapeutics: a potent therapeutic approach against BC

In the last decade, RNA technologies have demonstrated their potential in a wide range of applications [121–123]. Recently, mRNA-based vaccines have successfully become a promising substitute for traditional treatments, due to their high immunogenicity and safety profile. Furthermore, the production processes are low-cost and can be easily scaled-up [124]. Data obtained from population-wide administration of anti-SARS-CoV-2 mRNA vaccines have confirmed tolerability and safety of RNA based technology in the clinical field, paving the way to extend its use both in diagnostics and therapeutics, being particularly suited for personalized medicine strategies [125].

RNA therapeutics include different approaches based on diverse mechanisms and implied molecules like messenger RNAs (mRNA), antisense oligonucleotides (ASOs), aptamers, etc. This perspective focuses on two therapeutic strategies: (1) RNA interference (RNAi) and (2) messenger RNA (mRNA) [126]. RNAi is a post-transcriptional regulation of gene expression, initially discovered in plants [127]. In this process, short, non-coding RNAs bind the target endogenous RNA, by altering the transcript processing. In contrary, mRNAs are long, coding sequences, that encode protein products. Once introduced in the cytoplasm, mRNAs can produce coded protein by exploiting the intracellular translation machine. Thus, as therapeutic agents, they are administered to produce peptides or proteins in order to replace defective proteins or presenting antigens for vaccination [128, 129].

Modulating the expression of genes involved in infections, as in the pathogenesis of genetic diseases like cancer, opens a range of therapeutic opportunities: mRNA-based anti-cancer vaccines are already proving effective for cancer immunotherapy, while RNAi proved a powerful tool for targeted gene silencing in cancer gene therapy [130]. RNAi through small interfering RNAs (siRNAs) shows advantages in patients who are resistant to classic treatments [131, 132] and consequently is particularly suitable in personalized medicine.

Despite its potential, clinical applications of RNAi are still limited by a series of critical issues: (i) being negatively charged, RNAs are not able to cross cell membranes; (ii) they are highly unstable in the systemic circulation, where they are easily degraded by enzymes like serum RNases and endonucleases; and (iii) their therapeutic use is affected by off-target effects. So far, different strategies have been explored to overcome these limitations, for example, chemical modifications, conjugation to carbohydrates, and inclusion in liposomal particles have all been proved to increase selective targeting and prevent RNA degradation. Moreover, NPs have also proved an effective system to overcome these limitations and have shown to be great nano-carriers for stable RNA delivery. Among them, LNPs have been largely proven to be excellent RNA carriers, showing several advantages, as described in the "The effect of structure, composition and surface modification on delivery efficacy" section. In this part the most recent advances on RNAi technology will be discussed, with a focus on LNPs application as effective nanocarriers for RNAs [133].

### Recent advances in microRNA technology and clinical applications

MicroRNAs (miRNAs) are endogenous, single stranded, non-coding RNAs of about 22 nucleotides in length, which function at post-transcriptional level as modulators of gene expression. Over 2500 human miRNA sequences have been identified so far to regulate mammalian gene expression. Additionally, over 60% of human protein-coding genes have been found to contain at least one binding site for miRNA, demonstrating the significant contribution of miRNA-mediated regulation in the human genome [134]. miRNAs are key regulators of several pathophysiological processes, including cellular proliferation, and differentiation [135]. Several miRNAs are exclusively expressed or downregulated in certain cancers. It has been proven that miRNAs can act as oncogenes (oncomiRs) or onco-suppressors. Moreover, they are used as therapeutic agents to silence overexpressed genes and to revert the phenotype in cancer cells. Several studies have focused on targeting cancer by either inhibiting oncomiRs or overexpressing tumor suppressor miRNAs as a promising cancer treatment strategy [136]. Among the studied suppressive miRNAs, miR-34a has been proven to have strictly suppressive features in different cancer types [137]. Additionally, miR-34a is involved in the regulation of drug-resistance in BC, both in vitro and in vivo. Down regulation of miR-34a has been reported in MDR-MCF-7 cells compared with MCF-7 cells. Moreover, transfecting miR-34a mimics into MDR-MCF-7 cells led to partial MDR reversion. Additionally, by comparing the expression levels of miR-34a in a cohort of 113 patients with BC, the authors demonstrated the low expression of miR-34a is related with poor both overall and disease-free survival [138]. Another suppressive miRNA, miR-200c, is downregulated in BC tissues and cell lines, and its low expression is associated with poor prognosis [139]. In the case of oncogenic miRNAs, miR-182-3p is significantly upregulated in TNBC tissues and cell lines, indicating its potential oncogenic role in breast cancer [140].

Although some issues have still to be addressed, over the last decade the development of miRNA therapeutics has significantly improved demonstrating that nanotechnology can lead to clinical application [141].

#### LNPs for miRNA delivery

LNPs are effective delivery agents for miRNA molecules. In the case of BC, several studies have been performed to combine LNPs with miRNA as displayed in Table 2. In a pioneering study, Liu et al*.* developed a LNPs-based system for the simultaneous delivery of paclitaxel (PTX), and miR-200c [142]. Another study by Hayward et al*.* used LNPs to deliver miRNA125a-5p to 21MT-1 cells silencing HER2. They coated LNPs with hyaluronic acid as targeting agent. This improved the targeting of cancer cells, endosomal escape, and homogenous dispersal of miRNA in the cytoplasm. Additionally, the HER2 proto-oncogene was knocked down at both the transcriptional and translational levels, as demonstrated by qRT-PCR and western blot analyses [143].

**Table 2:** Recent studies on LNPs as nanocarrier for RNA delivery in breast cancer therapy

**Formulation**	**RNA therapeutic**	**Cargo**	**Main results**	***In vitro model***	***In vivo model***	**Dose used**	**Ref.**
DOTAP, DSPC, cholesterol and DMG-PEG	Cas9 mRNA/ gRNA	-	Impaired migration, and invasion capacity of cancer cells *in vitro*. reduced migration and invasion capacity of cancer *cells in vivo*.	MDA-MB-231	Nude mice	1 mg/kg	[173]
SM-102, cholesterol, DSPC, DMG-PEG2K and DSPE-PEG2K-PEP	pTEN mRNA	-	Autophagy-mediated cell death in 4T1 tumors, resulting in effective anticancer immune responses.	CT26, CL25, 4T1, 4T1-Luc, HCC1937	4T1-Luc	0.6mg/kg On days 5;8;11;14.	[174]
Ionizable SS-OP lipid, DOPC, cholesterol and DMG-PEG2000	uniSTING mRNA	-	Intratumoural or systemic injection of results in potent antitumour efficacy across established and advanced metastatic tumour models, including triple-negative breast cancer,	4T14T1-Luc2B16-F10Hepa1-6、ES-2THP-LLC1 and HEK293T	4T1-Luc2 tumour-bearing mice	15μg/kg On days 0;3;6;9.	[172]
Phosphatidylcholine (PC), Monostearin, oleic acid	mir-200c	PTX	miR-200c delivered by LNPs enhanced the effects of PTX in MCF-7in	MCF-7	-	10-400 mg/ml	[146]
PC, 1,2-Dipalmitoyl-sn- Glycero-3-Phopshoethanolamine (DPPE), and Cholesterol (CHOL)	miR125a-5	-	LNPs-miRNA125a-5p gave knockdown of the HER2.prolReduced proliferation, and migration.	21MT-1	-	-	[147]
Distearoylphosphatidylcholine (DSPC)/CHOL/ 1,2‐dioleyl‐3‐dimethylammonium propane (DODAP) / N‐palmitoyl‐sphingosine‐1‐{succinyl[methoxy(polyethylene glycol)2000]} (PEG2000‐Cer16)	MiR-182-3p	-	LNPs -miR‐182‐3p reduced tumor volume in vivo in various TNBC models .	HeLa,HCT116, Saos-2, U2-OS, MDA-MB-231, MDA-MB-436 and BJ. MDA-MB-436	CB17-SCID (CB17/Icr-Prkdcscid/IcrIcoCrl, 6 weeks old) female mice	**20 μg/mlmouse** at days 5;8;12;15;19;22.	[148]

In a recent study, Dinami et al*.*, utilized the miRNA-based strategy to inhibit the telomeric protein Telomere repeat binding factor 2 (TRF2), by inducing cancer cell death in TNBC. The proposed LNPs’ composition consisted of a lipid mixture of DSPC/CHOL/DLODAP/PEG_2000_‐Cer_16_ (25/45/20/10 w/w). This system reduced TRF2 levels in HeLa, HCT116, Saos-2, U2-OS, MDA-MB-231, MDA-MB-436 and MDA-MB-436 growth. It was found that the TRF2 silencing by miR‐182‐3p led to DNA damage at the telomere and pericentromeric sites, thus resulting in genomic instability. Moreover, the study demonstrated that using LNPs to encapsulate miR‐182‐3p decreased tumor volume in vivo in multiple TNBC models, including the Olaparib‐resistant tumor xenografts derived from the patients. Lastly, LNPs‐miR‐182‐3p were also able to cross the blood–brain barrier, eliciting their potential against brain metastasis [144].

**Fig. 6:**
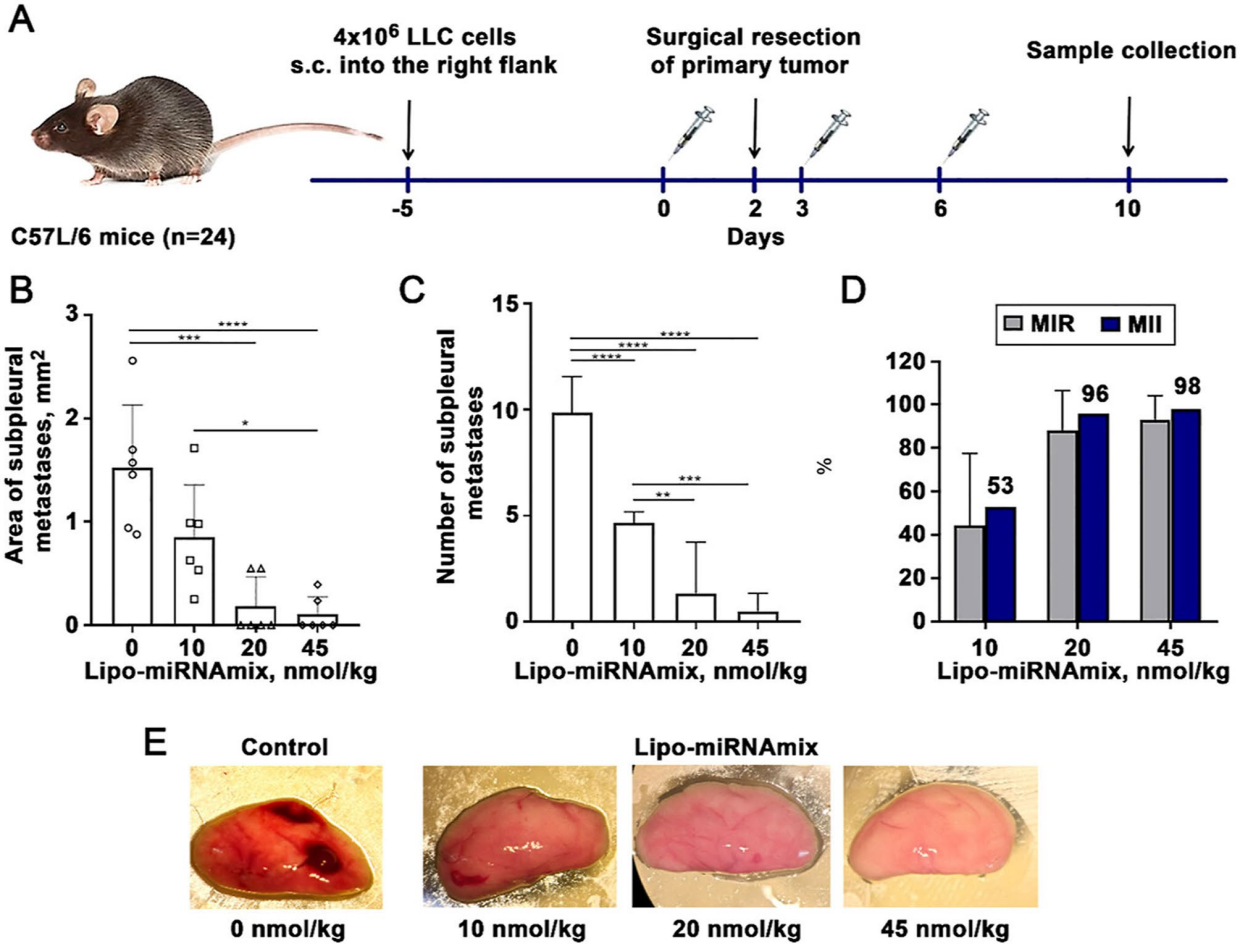
Impact of three miRNAs (miR-195–5p/miR-520a/miR-630) encapsulated in LNPs on the formation of metastasis in the lungs of LLC-bearing mice. (**A**) Cartoon depiction of the workflow; s.c.: subcutaneous injection. (**B**) The area and (**C**) number of subpleural metastases in the lungs of mice after three-time injection of Lipo-miRNAmix in a dose of 10, 20, or 45 nmol (RNA)/kg. (**D**) The MII and metastasis inhibition rate (MIR) in the three experimental groups of mice. (E) Representative images of a lung excised from mice of the different groups. Data were subjected to one-way ANOVA with post hoc Tukey’s test. Data are presented as mean ± SD *p < 0.05; **p < 0.01; p < 0.001; **** p < 0.0001. Reprinted with permission from ref. [145]

Nevskaya et al. proposed a LNP encapsulating three different microRNAs, namely miR-195 − 5p, miR-520a, and miR-630. These miRNAs downregulated the expression of stemness genes such as SOX2, MYC, TERT, and FZD9 in human breast cell lines. The system was intended to prevent metastasis by inhibiting the dedifferentiation of CD44 − cancer cells. Data show that the system effectively inhibited the ability of cells to form mammospheres in vitro and prevented lung metastasis in a C57L/6 mouse model (Fig. 6) [145].

### Recent advances in siRNA technology and clinical applications

siRNAs, approximately 25 nucleotides in length, belong to a class of double-stranded RNAs that act as mediators in the RNAi. After being processed by dedicated enzymes in the cytoplasm, siRNAs bind a sequence of a complementary mRNA by inducing its degradation and preventing the translation in the corresponding protein product. To date, four siRNA-based treatments have been approved by FDA, demonstrating the potential efficacy and safety of this therapeutic approach [146]. Particularly, RNAi through siRNAs appears to be a promising tool to perform a selective gene silencing in the context of targeting tumor-specific mRNAs [84, 147]. Unlike microRNAs, siRNAs only bind to a target mRNA sequence, reducing the off-target effect that often leads to the silencing of multiple genes. Despite this, certain aspects, like degradation prevention and effective transportation of the siRNA still pose existing challenges. As mentioned above, these limitations are partially solved by using LNPs as carriers [148].

#### LNPs for siRNA delivery

Over the last decade, siRNAs have emerged as potent therapeutics against cancer due to their ability to silence genes involved in tumorigenesis and metastasis [149]. However, the delivery of siRNAs to the target remains a major inhibition [150]. LNPs have emerged as a suitable tool for an effective delivery of siRNAs; LNPs protect the siRNA from degradation and favour a selective delivery to the target cells, by increasing their bioavailability and reducing the off-target effect. Therefore, numerous studies have focused on LNPs as siRNAs delivery systems for therapeutic applications [151–155]. In 2018, the results of a clinical trial based on the use of a siRNA drug, Patisiran, were published, showing breakthrough results in the field [57]. The drug was used to treat patients suffering from hereditary transthyretin (TTR) amyloidosis. The disease is caused by the accumulation of misfolded TTR as amyloid fibrils in various tissues, including the heart, nerves, and gastrointestinal tract. Interestingly, the corresponding mRNA is primarily expressed in the liver. Thus, Adams et al. used a LNP nano-carrier to deliver siRNA to the liver, successfully inducing the degradation of mRNA encoding for TTR via RNAi [156]. Patisiran is currently present on the market as the first siRNA drug FDA approved. Recently, Kiaie et al*.* formulated siP2X7-LNPs, a lipid nano carrier for the delivery of siRNA targeting P2X7 receptor (P2X7R) [157]. The system included a novel symmetric branched ionizable lipid (SIL), able to deliver the loaded siRNA, and to target P2X7R in mouse mammary carcinoma cells (4T1). The findings suggest that SIL effectively transfected siP2X7, and siP2X7-LNPs notably suppressed migration by inducing apoptosis in 4 T-1 cells. These data demonstrated therapeutic efficacy of siRNA drugs in a triple-negative BC cell line.

**Fig. 7:**
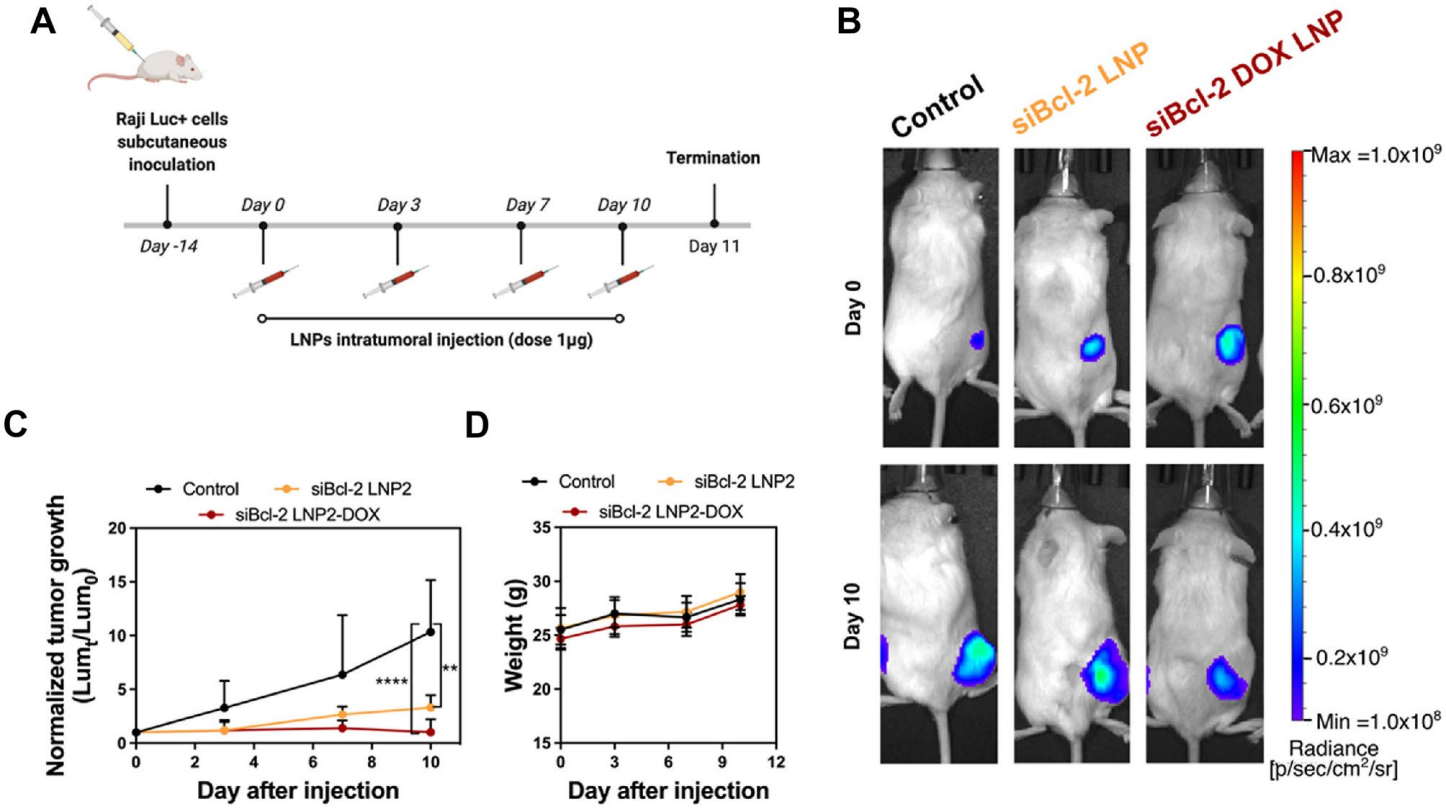
In vivo delivery of DOX-conjugated siRNA LNPs to treat a mouse model of lymphoma (**A**) Drug treatment schedules for *in vivo* experiments. The treatment doses for Bcl-2 siRNA and DOX were 1 μg and 0.1 μg per mouse, respectively (**B**) Representative bioluminescence images of tumor-bearing mice (**C**) Tumor growth curves for different treatment groups. All data is normalized to bioluminescence radiant efficiency (photons/sec/cm2/sr) on day 0. Data are plotted as mean ± SD, *n* = 5; ∗∗ *P* < 0.005, ∗∗∗∗ *P* < 0.0001 *vs*. Control (**D**) Average body weight of different treatment groups during the treatment schedule. Reprinted from ref. [158] under the CC BY-NC-ND license

To achieve synergistically enhanced tumour treatment effects, co-delivery of chemotherapeutic drugs and siRNA represents a promising opportunity. However, incorporating the drugs into siRNA-LNPs may disrupt their structure and provoke low encapsulation efficiency. To address those issues, in 2023 Butowska et al*.* proposed a surface modified siRNA-loaded LNP, obtaining a potent knockdown of Bcl-2 transcript in vitro and in vivo experiments. These authors used a sulfur-containing phospholipid to conjugate a (6-maleimidocaproyl) hydrazone derivative of DOX (DOX-EMCH) on the surface of the LNPs. They showed a successful delivery of DOX-EMCH to the cell nucleus while achieving potent knockdown of Bcl-2 in Raji cells. In vivo studies, conducted in Raji Luc + tumor-bearing NSG mice, further demonstrated the effectiveness of this system in inhibiting tumor growth. As shown in Fig. 7, they intratumorally injected different formulations, and they demonstrated a reduction in mice treated with siBcl-2 DOX LNP with the respect the used controls.

These findings suggest that LNPs conjugated with doxorubicin could serve as a platform for co-delivering nucleic acids and chemotherapy drugs in combination cancer therapies [158]. Since overexpression of Bcl-2 occurs in breast tumour leading to increased survival rate, the investigated system was presented as a potential tool for BC therapy [159]. Based on the discussed results it is expected that the LNPs-siRNA technology will be increasingly applied for BC treatment and have outlets in clinical practice with enormous benefits for the patients.

### Recent advances in mRNA technology and clinical applications

mRNA is a single strand nucleic acid sequence, and it is the molecule resulting from the transcription of a template strand of DNA. As mature transcript, it carries genetic information from the nucleus to the cytoplasm, where it is translated by ribosomes, leading to proteins’ synthesis. Due to its physiological function and properties, mRNA can be exploited as therapeutics, by producing proteins whose function is lost or whose production is deficient, depending on the disease [160].

Additionally, mRNA is easily designed, exhibiting minimal off-target effects, and compared to antibodies or proteins, it is considerably simpler to purify and scale up. [161, 162]. Also, mRNA activity is confined to cytoplasmatic compartment, and its expression is transient, dramatically decreasing the possibility to cause genetic mutations or integration processes. During the last pandemic the potential application of mRNA as nucleic acid became clear, and data collected following its administration confirmed its robustness and safety. This paved the way for exploring new applications of mRNA therapeutics.

For example, Karikó, Weissman, and co-workers developed approaches for modifications in the nucleotides that incrementally increased the stability, immunogenicity, and translation efficacy of mRNA [163]. All these attributes display the strong potential of mRNA to be used not just in vaccines but also for targeting cancer cells.

In cancer therapy, mRNAs encoding for tumour suppressor proteins antigens or cytokines are transfected to inhibit cancer cells proliferation, or enhance the immune response. Moreover, mRNA-encoded genome-editing proteins can destroy targeted genes by improving the treatment efficacy [122]. LNPs have also been proven to be the most efficient platform for mRNA delivery.

#### LNPs for mRNA delivery

Advances in drug delivery systems have contributed to the pre-clinical development of mRNA therapeutics, proposing them as a new class of drugs [107]. Out of a variety of materials like lipids, lipid-like polymers, proteins, etc*.*, LNPs have emerged successfully in clinics for the delivery of mRNAs [164, 165]. To evaluate their efficacy in vitro and in vivo, El-Mayta et al*.* recently demonstrated the mRNA encapsulation efficiency to be 92.3% for C12-200 mRNA-LNPs with a molar ratio of 35/16/46.5/2.5 (ionizable lipid: helper lipid: cholesterol:lipid-PEG) [166]. This gives insights into the promising perspectives on the use of LNPs as mRNA carriers.

A study by Zhang et al*.* developed PTX amino lipid (PAL) derived NPs to integrate both chemotherapeutics and p53 mRNA to develop new treatment approaches for TNBC. The PAL p53 mRNA LNPs were found to show efficient PTX loading capacity (94.7% ± 6.8%) and mRNA encapsulation efficiency (88.7% ± 0.7%), in comparison to clinically used drugs like Abraxane® and Lipusu®. The LNPs also demonstrated the cytotoxicity of PTX and p53 mRNA in TNBC cells. Additionally, in vivo*,* anti-tumor efficacy was elicited in the TNBC mouse models. This provides a new platform for integrating chemotherapy with personalized medicine for treating TNBC [167].

Another in vitro study from Nakashima et al*.* aimed at developing a novel treatment strategy for refractory BC. It combined the tumour-tropic LNPs with inducible caspase9 (iC9) mRNA. The study demonstrated that the LNPs efficiently entered BC cells by delivering the mRNA cargo. Therefore, cytotoxicity mediated by LNP-mRNA was observed on three different BC cell lines (MDA-MB231, SKBR3, and MCF-7) loaded with iC9 mRNA. These effects resulted from iC9-mediated apoptosis through the activation of downstream caspase-9 and caspase-3/7 [168].

**Fig. 8:**
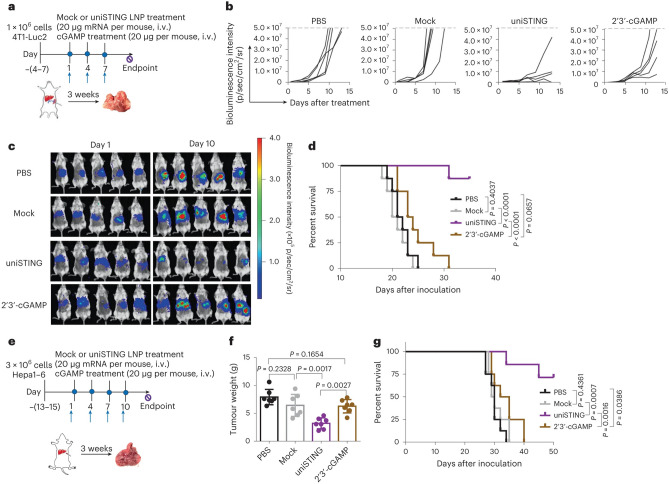
The results of systemic uniSTING treatment exerting potent antitumor effects on orthotopic/metastatic tumours. **a**, Treatment scheme for the 4T1-Luc2 liver metastatic tumour model with the indicated formulations. **b**, Spider plots of individual tumour growth curves measured by bioluminescence intensity. n = 5. **c**, In vivo bioluminescence imaging of mice bearing liver metastatic tumours on days 1 and 10 following treatment. n = 5. **d**, Kaplan–Meier survival curves of mice treated with indicated formulations. n = 8. **e**, Treatment scheme for orthotopic Hepa1-6 HCC tumour-bearing mice with the indicated formulations. **f**, Average tumour weight in HCC tumour models after indicated treatments. n = 7. **g**, Kaplan–Meier survival curves of mice treated with indicated formulations. n = 8. For survival studies, 5 × 10^7^ of bioluminescence intensity was used as the endpoint criteria in the 4T1 liver metastatic tumour model and a 30% weight loss was used as the endpoint criteria in the HCC tumour model. Each line represents one survival curve for each group; log-rank (Mantel–Cox) test. Significant differences were assessed using a one-way ANOVA and Tukey’s multiple-comparisons test. Results are presented as mean ± s.d. Reprinted with permission from ref. [169]

Recently, Wang et al*.* developed a universal stimulator of interferon genes mimic (uniSTING) based on a polymeric architecture, capable of activating STING signalling in various mouse and human cell types. Unlike traditional STING agonists, uniSTING selectively stimulates pathways associated with tumour control while avoiding tumour progression. Additionally, uniSTING enhances dendritic cell maturation and antigen-specific CD8 + T-cell responses, further bolstering its therapeutic potential. Extracellular vesicles (EVs) released from uniSTING-treated tumour cells contribute to dendritic cell sensitization, while combination therapy with α-Wnt2b antibodies shows synergistic inhibition of tumour growth and prolonged animal survival (Fig. 8).

Delivery of uniSTING-mRNA via LNPs demonstrated potent efficacy in diverse tumours, including TNBCs, LLC1, B16-F10 and advanced orthotopic/metastatic liver tumour models surpassing existing STING agonists. Intravenous administration of uniSTING drastically inhibited 4T1 liver metastatic tumour growth and eradicated metastatic tumours in 40% of mice compared with the 2′3′-cGAMP-treated group which showed negligible effect (Fig. 8a–d). Moreover, mice with the orthotopic Hepa1-6 liver cancer exhibited reduced tumour burden and prolonged survival following intravenous injection of LNP-uniSTING-mRNA than controls (Fig. 8e–g). Overall, these findings highlight the promising role of LNP-mediated delivery of uniSTING-mRNA in overcoming therapeutic barriers associated with STING agonist therapy, particularly in cancers with downregulated or absent STING expression [169]. Table 2 summarises the most recent studies on LNPs for RNAs delivery in BC treatment.

## Conclusions and future perspectives

BC is the most common cancer in women. Being a heterogeneous disease, different therapeutic regimens are currently used, depending on BC subtype, staging, and patients’ compliance. However, current available treatments are not in all cases effective; particularly, TNBC is the less responsive subtype. Hence, patients can experience recurrences over time, with serious health complications. Recently the scientific community has made considerable efforts to find more efficient approaches. By exploiting nanotechnology and RNA-based therapies, promising alternatives for the treatment of BC have been developed.

LNPs have shown great potential as delivery platform: they are, indeed, easily prepared and purified, and they can be smoothly scaled-up. LNPs are successfully used as nanocarriers in immunotherapy applications, proving their safety in clinical use. Furthermore, their surface can be easily modified with targeting molecules like peptides or antibodies to enhance the selectivity, the therapeutic effect, by increasing their bioavailability and decreasing the off-target effect and the effective dose.

Moreover, LNPs are the most effective delivery platform for RNAs, as largely testified by the worldwide administration of mRNA anti-COVID vaccines. Additionally, LNPs encapsulating RNA cargos have been proven effective in BC treatment [170–173], both in vitro and in vivo, demonstrating efficacy also for the treatment of metastatic forms of BC [174]. However, more clinical studies would be required to better understand the potential clinical applications and the safety profile of these systems applied to BC therapy.

The results of the last few years emphasized by the extraordinary outputs recorded after the worldwide administration of mRNA anti-COVID vaccines, confirm that the therapeutic potential of LNPs, combined with RNA technologies, will reach outstanding result in cancer gene therapy, including neurological and skin cancers [175–178].

## Data Availability

Data sharing not applicable to this article as no data set was generated or analyzed during the current study.
